# Preventive Care Use among the Belgian Elderly Population: Does Socio-Economic Status Matter?

**DOI:** 10.3390/ijerph110100355

**Published:** 2013-12-24

**Authors:** Sarah Hoeck, Johan van der Heyden, Joanna Geerts, Guido van Hal

**Affiliations:** 1 Department of Epidemiology and Social Medicine, Faculty of Medicine and Health Sciences, University of Antwerp, Universiteitsplein 1, Antwerp 2610, Belgium; E-Mail: guido.vanhal@uantwerpen.be; 2 Scientific Institute of Public Health, Julliette Wytsmanstraat 14, Brussels 1050, Belgium; E-Mail: johan.vanderheyden@wiv-isp.be; 3 Social Security Research Group, Federal Planning Bureau, Kunstlaan 47-49, Brussels 1000, Belgium; E-Mail: jg@plan.be

**Keywords:** preventive care, socioeconomic status, elderly population, Belgium

## Abstract

*Objective*: To analyze the association between influenza and pneumococcus vaccination and blood cholesterol and blood sugar measurement by Belgian elderly respondents (≥65 years) and socio-demographic characteristics, risk factors, health status and socio-economic status (SES). *Methods*: A cross-sectional study based on 4,544 non-institutionalized elderly participants of the Belgian Health Interview Surveys 2004 and 2008. Multivariate logistic regression models were constructed to examine the independent effect of socio-demographic characteristics, risk factors, health status and SES on the four preventive services. *Results*: After adjustment for age, sex, region, survey year, living situation, risk factors (body mass index, smoking status, physical activity) and health status (self-assessed health and longstanding illness) lower educated elderly were significantly less likely to report a blood cholesterol and blood sugar measurement. For instance, elderly participants with no degree or only primary education were less likely to have had a cholesterol and blood sugar measurement compared with those with higher education. Pneumococcus vaccination was not related to educational level, but lower income groups were more likely to have had a pneumococcus immunization. Influenza vaccination was not significantly related to SES. *Conclusion*: The results highlight the need to promote cholesterol and blood sugar measurement for lower SE groups, and pneumococcus immunization for the entire elderly population. Influenza immunization seems to be equally spread among different SE groups.

## 1. Introduction

With the ageing of the population, maintaining health in old age will become increasingly important. Health is unevenly distributed across the European society, and lower socioeconomic status (SES) is associated with higher morbidity and mortality rates [1]. Socioeconomic inequalities in health may be due to differential uptake of preventive care. While primary prevention (immunization) could prevent health problems, secondary prevention or early diagnosis (blood cholesterol and blood sugar measurement) gives opportunities for better treatment. Literature reveals inequities in health service utilization [2,3] and in preventive care use [4,5,6,7]. Prevention is one of many strategies to achieve healthy ageing [8]. Preventive care can improve the survival and well-being of the elderly people significantly, and helps controlling healthcare spending [9]. While in curative care the goal is usually to restore patients health, in preventive care the goal is to shift the entire population to a healthier level [10]. Ensuring the health of a population is more difficult than delivering healthcare to an individual and requires focusing on health inequalities [10]. Representative studies concerning possible inequities in preventive care use are needed to reinforce healthy ageing in Europe. Despite the importance of preventive care in early identification of risk factors and preventing illnesses, there is evidence of inequity in preventive care use within European countries, favouring higher SE groups [4,11,12,13,14]. Lower SE groups use significantly less preventive care than higher SE groups [14,15,16]. Those who have the greatest benefit from the services seem to be less likely to use them [17]. Differential uptake therefore has the potential to exacerbate health inequalities [18]. Among the reported studies the influence of SES is somewhat controversial. While influenza and pneumococcus immunization have been shown to be cost-effective in reducing and preventing morbidity and mortality among the elderly population [19,20,21], there is still evidence of inequity in uptake in European countries. Some studies are in agreement that lower SE groups have a lower uptake of influenza and pneumococcus immunization [13,14,15,18,20,21,22,23], while other studies indicated that lower SE groups are more likely to be vaccinated [23,24,25]. Although cholesterol and blood sugar monitoring allow the detection of many forms of illnesses like cardiovascular diseases (CVD) or diabetes [26], an increasing body of evidence has revealed inequity in the use of such preventive services, mostly favouring higher SE groups [4,14,27,28,29]. A study of 13 European countries, including Belgium, investigating income-related inequalities in preventive care, found that richer individuals of 50 years and older were more likely to do blood tests despite their lower diagnostic needs for that care [14], whereas Patel [11] found no association between SES and cholesterol measurement. Socio-demographic factors affecting preventive care use were discussed with controversial conclusions in the literature. In general, attenders at preventive services are older than non-attenders [7,18,30], although some studies found no associations between age and attendance [18]. Among elderly people, an age-related trend to higher immunization rates [21,25] and general health checks is documented [7]. Other studies find a negative relationship between age and prevention [31]. Studies consistently indicate that males are less likely to use preventive care [7,18,32]. Living situation affects influenza immunization and general health checks, indicating that attenders were more likely to be married or cohabiting [18,25,33,34]. Those who have a partner may be reminded of the importance of general health checks or vaccination. They also may be worried about transmitting diseases to their partner [15].

The use of preventive services is often associated with certain risk factors. For instance, smokers and obese people are less likely to have general health checks [18]. Smoking and engaging in regular exercise are significant factors that affect influenza immunization [7,33,34]. Preventive care use is also driven by the health status of an individual. A poor health status positively affects preventive care use [16]. Elderly with chronic conditions, e.g., heart and lung diseases—are most likely to take-up vaccination [15,32]. An example of unobservable risk factors are the “worried well”, people who are worried about getting ill and see a doctor when it is not necessary. In this case, the use of preventive services is associated with the lack of certain risk factors. A study about CVD prevention in primary care found that the worried well were more likely to participate in primary care prevention programs, while high-risk patients are less interested in reducing their risk factors [35].

In Belgium influenza and pneumococcus immunization is recommended for individuals who have either an increased risk to develop severe influenza disease or pneumonia (*i.e.*, persons aged ≥ 65 years, and persons with certain chronic medical conditions) or who are likely to transmit the virus to vulnerable groups (e.g., health care workers) [36]. Regular cholesterol and blood sugar checks are recommended for persons aged ≥40 year and for individuals with an increased risk. To date little attention has been paid to SE gradients (income and educational inequalities) in blood cholesterol, blood sugar measurement and influenza and pneumococcus immunization by the elderly Belgian population. The purpose of this study is therefore to explore the existence of such a SE gradient, in the use of these four preventive services by using data from a representative population based on the Belgian Health Interview Survey (HIS), and to check whether Belgian health policy succeeds in guaranteeing an equal distribution of preventive services among elderly persons with equal needs. In this study we also determine which predisposing, enabling and need factors are associated with preventive care use.

## 2. Methods

### 2.1. Survey Data: The Belgian Health Interview Survey

This cross-sectional study was based on pooled data (2004 and 2008) of the Belgian Health Interview Survey (HIS) in which a representative sample of the Belgian population of 15 years and over was interviewed about their life style, health status, and healthcare utilisation. Participants were selected according to a multistage sampling design, including stratification, clustering and systematic sampling [37]. The participation rate of the HIS 2004 was 61.4%, yielding a sample group of 12,650 respondents, and 55.0% in HIS 2008, yielding a sample group of 11,254 respondents. In this study, statistical analyses were restricted to non-institutionalised elderly people (aged ≥ 65 years, n = 4,544). Elderly respondents with diabetes were excluded. Proxy interviews were excluded, because the variable “self-assessed health”, a crucial dimension of health status, was not available in these cases. A more detailed description of the study population is presented in Table 1, under the paragraph “Results”. 

### 2.2. Preventive Care Use

Preventive services investigated, all dichotomous (yes/no), were pneumococcus immunization in the past 5 years, influenza immunization in the past year, blood cholesterol measurement in the past 5 years and blood sugar measurement in the past 3 years. 

### 2.3. Socio-Economic Status

Equivalent household income and highest educational level within the household were included to measure SES. A higher household income increases financial accessibility of public and private healthcare services and is therefore a crucial enabling factor. We used the equivalent household income instead of the individual’s income as differences between the income of household members can be rather large, and the SES of an individual is often based on the household income. Household income refers more to the real availability of economic means within the family, while individual income rather refers to a measure of status, prestige, and power [38]. Equivalent household income (a standardized net monthly income) takes into account household size and composition using the modified Organisation for Economic Co-operation and Development (OECD) scale (which assigns a value of 1 to the head of the household, 0.5 to each additional adult member and 0.3 to each child) [39] and measures income from work and allowances (including pension, but not wealth, properties, rent, interests or other indicators of financial capacity), which is recoded into the following categories: <€750, €750–€1000, €1000–€1500, €1500–€2500, and >€2500.

Education is an important enabling factor, as it influences the course of life in several domains (working conditions, living standard, lifestyle), and is related to problem-solving skills, general and health-related knowledge, health beliefs, and health behaviour. The impact of educational level continues during later life. We used the highest educational level within the household, as lower educated household members could benefit from the presence of a higher educated person [40]. Highest level of education within the household is recoded into the categories no degree or primary education, lower secondary, higher secondary and higher education. Given the independent effects and unique features of education and income, a combined use of these indicators is preferable [41]. The use of education as a proxy for income (or *vice versa*) [38] cannot be justified. Previous research [42] indicates that combining SES indicators yields stronger gradients than using a single measure. 

### 2.4. Socio-Demographic Characteristics, Health Status and Risk Factors

Although there might be no SE gradient in the use of cholesterol and blood sugar measurement and influenza and pneumococcus immunization, the incorporation of health status and risk factors could reveal a significant inequity in the use of such preventive care. Preventive services were analyzed with adjustment for socio-demographic characteristics: age, sex living situation (alone or cohabitant), and region (Flemish region, Brussels capital region, Walloon region). In Belgium, preventive care belongs to the responsibilities of the regional governments, thus region could affect preventive care use. We included self-assessed health and longstanding illness or health problems as need factors. “Self-assessed health” was evaluated from the answers to the question “How is your health in general?”, which were recoded into two categories: “good to very good health” and “moderate, bad to very bad health”. Longstanding illness or health problems was based on the question “Do you have any longstanding illness or longstanding health problem? Yes or no”. We also included risk factors that may influence the preventive care use: body mass index (BMI) (recoded into <18.5, 18.5–25, 25–30 and 30+); smoking status (recoded into never smoked, former smoker, occasional or daily smoker) and physical activity (recoded into two categories ≥30 min of physical activity per day and <30 min of physical activity per day).

### 2.5. Statistical Methods

Multivariate logistic regression models were used to examine the independent effect of socio-demographic characteristics, risk factors, health status and SES on preventive care use. In a first model we assessed the effect of each characteristic on the four preventive services after adjustment for age and gender. In a second model we explored SE gradients in the use of preventive services after adjustment for socio-demographic characteristics, risk factors and health status. The sample size in the model 1 and 2 is based on all cases with complete data concerning all the variables used in the analysis (n = 1,649). Adjusted odds ratios (ORs) with 95% confidence intervals (CIs) were calculated. All analyses were carried out in Stata-MP, version 10. 

## 3. Results

The characteristics of the study population are shown in Table 1. In the past 5 years before the survey 13.4% have had a pneumococcus immunization, and 72.0% a blood cholesterol measurement. In the past 3 years before the survey 55.3% have had a blood sugar measurement and in the past year before the survey 63.1% have had an influenza immunization. 

Table 2 shows the results (odds ratio’s) of the regression models of preventive care use (yes or no) according to socio-demographic variables, health status, risk factors and SES, after adjustment for age and sex.

*Pneumococcus immunization—*Pneumococcus immunization varies according to gender, region, living situation, longstanding illness or health problem, BMI and SES. Women, elderly living alone and elderly with no degree or only primary education as highest educational level within the household are significantly less likely to have had a pneumococcus immunization. Elderly respondents living in the Brussels capital and Walloon region are more likely to have had a pneumococcus immunization than the elderly living in the Flemish region. Elderly respondents with a longstanding illness or health problem and elderly with a BMI <18.5, elderly with an equivalent household income of €1500**–**€2500 are significantly more likely to report pneumococcus immunization. 

**Table 1:** Characteristics of the study population (absolute figures and weighted **^1^** percentages).

Characteristic	Number of Participants	Percentage of the Total
**Total**	4,544	100.0
**Year of the Survey**		
2004	2,513	48.6
2008	2,031	51.4
**Age, mean (SD)**	4,544	77.3 (0.12)
**Age (years)**		
65–74	1,958	57.4
75–84	1,442	35.7
≥85	1,144	6.9
**Sex**		
female	2,690	57.2
male	1,854	42.8
**Living situation**		
cohabitant with other(s) in a home situation	2,249	60.1
living alone	1,910	32.6
no information	385	7.3
**Place of residence**		
Flemish region	1,751	61.7
Brussels Capital region	1,111	8.0
Walloon region	1,682	30.3
**BMI**		
no information	324	6.1
<18.5	160	2.5
18.5–25	1,897	39.2
25–30	1,599	38.3
30+	564	13.9
**Smoking status**		
No information	913	13.9
Never	2,185	50.4
Former	1,015	24.9
Occasionally/daily	431	10.8
**Physical activity**		
No information	1,683	33.5
≥30 min/day	520	16.3
<30 min/day	2,341	50.2
**Self-assessed health ^2^**		
No information	744	10.5
Good to very good health	2,145	51.3
Moderate, bad to very bad health	1,655	38.2
**Longstanding illness or health problem**		
No information	815	12.0
No	2,110	50.6
Yes	1,619	37.4
**Highest level of education within the household**		
No information	156	2.9
No degree or primary education	1,377	28.7
Lower secondary	1,045	24.6
Higher secondary	1,091	26.2
Higher education	866	17.6
**Equivalent household income (€)**		
No information	699	14.4
<750	686	14.3
750–1,000	945	21.2
1,000–1,500	1,595	38.0
1,500–2,500	521	10.1
>2,500	98	2.0
**Blood cholesterol measurement in past 5 years**		
No information	77	1.6
Yes	2,883	63.1
No	1,584	35.3
**Blood sugar measurement in past 3 years**		
No information	1,256	22.1
Yes	2,378	55.3
No	910	22.6
**Influenza immunization in past year**		
No information	77	1.6
Yes	2,883	63.1
No	1,584	35.3
**Pneumococcus immunization in past 5 years**		
No information	596	14.0
Yes	653	13.4
No	3,259	72.6

Notes: ^**1**^ See reference [37]; **^2^** By answering the question “How is your health in general?”

**Table 2:** Use of preventive servicesby respondents 65 years and over (n = 1,649) according to sociodemographic variables, health and socioeconomic status, and after adjustment for age and sex.

Preventive Service	Pneumococcus Immunization (in past 5 years)	Influenza Immunization (in past year)	Blood Cholesterol Measurement (in past 5 years)	Blood Sugar Measurement (in past 3 years)
**Age**	1.01 (0.99–1.03)	1.04 (1.02–1.05) ***	0.99 (0.97–1.02)	1.01 (1.00–1.03)
**Gender**				
Female	0.72 (0.55–0.93) ***	0.83 (0.67–1.02)	1.24 (0.93–1.66)	1.20 (0.95–1.52)
Male	1.00	1.00	1.00	1.00
**Region**				
Flemish region	1.00	1.00	1.00	1.00
Brussels region	2.17 (1.55–3.03) ***	0.87 (0.66–1.13)	1.23 (0.85–1.77)	1.56 (1.15–2.12) **
Walloon region	1.61 (1.17–2.21) **	0.86 (0.68–1.09)	1.41 (1.00–1.98) *	1.36 (1.05–1.77) ***
**Year**				
2004	1.00	1.00	1.00	1.00
2008	0.93 (0.72–1.21)	1.15 (0.94–1.42)	1.70 (1.25–2.32) ***	1.62 (1.28–2.06) ***
**Living situation**				
with other(s) at home	1.00	1.00	1.00	1.00
living alone	0.56 (0.42–0.75) ***	0.80 (0.64–0.99) ***	0.85 (0.62–1.67)	1.09 (0.85–1.40)
**Self-assessed health**				
good to very good health	1.00	1.00	1.00	1.00
fair, bad to very bad	1.22 (0.94–1.59)	1.62 (1.31–2.01) ***	1.54 (1.13–2.11) **	1.28 (1.01–1.63) **
**Longstanding illness or health problem**				
No	1.00	1.00	1.00	1.00
Yes	1.41 (1.09–1.83) **	1.55 (1.26–1.91) ***	2.05 (1.50–2.81) ***	1.76 (1.38–2.24) ***
**BMI**				
<18.5	2.45 (1.23–4.89) **	1.01 (0.53–1.95)	0.45 (0.21–0.96) *	0.46 (0.24–0.88) *
18.5–25	1.00	1.00	1.00	1.00
25–30	1.24 (0.93–1.65)	1.47 (1.17–1.85) ***	1.10 (0.79–1.52)	1.17 (0.90–1.52)
30+	0.96 (0.62–1.48)	1.41 (1.02–1.95) *	1.13 (0.70–1.82)	1.00 (0.70–1.43)
**Smoking status**				
never	1.00	1.00	1.00	1.00
former	1.21 (0.88–1.67)	0.97 (0.75–1.26)	1.40 (0.96–2.04)	1.21 (0.90–1.63)
occasionally/daily	1.45 (0.95–2.20)	0.76 (0.54–1.07)	0.93 (0.58–1.49)	0.86 (0.59–1.25)
**Physical activity**				
≥30 min/day	1.00	1.00	1.00	1.00
<30 min/day	1.43 (1.00–2.04)	1.14 (0.88–1.48)	0.76 (0.51–1.13)	0.97 (0.71–1.31)
**Highest level of education within the household**				
No degree or primary education	0.65 (0.45–0.94) *	1.05 (0.78–1.40)	0.44 (0.28–0.67) ***	0.48 (0.34–0.69) ***
Lower secondary	0.81 (0.55–1.81)	1.07 (0.79–1.45)	0.59 (0.37–0.95) *	0.57 (0.39–0.82) **
Higher secondary	1.04 (0.73–1.49)	1.04 (0.77–1.40)	0.65 (0.41–1.05)	0.58 (0.41–0.83) **
Higher education	1.00	1.00	1.00	1.00
**Equivalent household income (€)**				
<750	2.69 (0.79–9.12)	1.31 (0.70–2.44)	0.78 (0.33–1.86)	0.80 (0.39–1.64)
750–1,000	3.06 (0.92–10.15)	1.21 (0.66–2.21)	1.05 (0.45–2.47)	0.99 (0.49–1.99)
1,000–1,500	3.10 (0.94–10.15)	1.74 (0.96–3.14)	1.35 (0.59–3.13)	1.05 (0.53–2.09)
1,500–2,500	4.04 (1.21–13.54) *	1.28 (0.67–2.40)	1.32 (0.54–3.22)	1.21 (0.58–2.51)
>2,500	1.00	1.00	1.00	1.00

Notes: Complete case analyses, after adjustment for age and gender; Odds ratios (ORs); 95% confidence intervals (CIs), and *p* values (Wald Chi-square test); * *p* < 0.05; ** *p* < 0.01; *** *p* ≤ 0.001.

*Influenza immunization—*After adjustment for age and sex, are elderly living alone less likely to have had an influenza immunization. Elderly respondents who assess their health as moderate, bad to very bad, who have a longstanding illness or health problem and have a BMI of 25–30 or 30+ are significantly more likely to report influenza immunization. Influenza immunization does not seem to vary according to SES. 

*Blood cholesterol measurement—*After adjustment for age and sex, elderly people living in the Walloon region, respondents of the HIS 2008, and those who assess their health as moderate, bad to very bad, and those who have a longstanding illness or health problem are significantly more likely to have had a blood cholesterol measurement in the past 5 years. Elderly with a BMI <18.5 and lower educated elderly are significantly less likely to have had a blood cholesterol measurement. 

*Blood sugar measurement*—Blood sugar measurement in the past 3 years differs according to region, survey year, health status, BMI and educational level. Elderly respondents living in the Brussels’ or Walloon region are significantly more likely to have had a blood sugar measurement in the past 3 years compared with inhabitants of the Flemish region. Respondents of the HIS2008 were more likely to report having blood cholesterol measurement compared with respondents of the HIS2004. Those who assess their health as moderate, bad to very bad, and those who have a longstanding illness or health problem are significantly more likely to have had a blood sugar measurement in the past 3 years. Elderly with a BMI < 18.5 and lower educated elderly are significantly less likely to have had a blood sugar measurement.

Table 3 displays the results (odds ratio’s) of the multivariate logistic regressions in which was adjusted for all of the independent variables to gain a view on the remaining impact of SES when controlling for all other determinants on preventive care use.

**Table 3:** Use of preventive servicesby respondents 65 years and over (n = 1,649) according to socioeconomic status after adjustment for sociodemographic variables, health status and risk factors.

Preventive Service	Pneumococcus Immunization (in past 5 years)	Influenza Immunization (in past year)	Blood Cholesterol Measurement (in past 5 years)	Blood Sugar Measurement (in past 3 years)
**Age**	1.02 (1.00–1.04) *	1.04 (1.02–1.06) ***	1.00 (0.98–1.02)	1.01 (0.99–1.03)
**Gender**				
Female	0.84 (0.61–1.16)	0.76 (0.59–0.97) *	1.56 (1.09–2.22) **	1.27 (0.95–1.68)
Male	1.00	1.00	1.00	1.00
**Region**				
Flemish region	1.00	1.00	1.00	1.00
Brussels’ region	2.08 (1.45–2.98) ***	0.86 (0.65–1.15)	1.12 (0.75–1.67)	1.43 (1.03–1.99) *
Walloon region	1.59 (1.14–2.20) **	0.80 (0.62–1.03)	1.38 (0.97–1.96)	1.27 (0.97–1.68)
**Year**				
2004	1.00	1.00	1.00	1.00
2008	0.90 (0.68–1.19)	1.10 (0.88–1.38)	1.54 (1.12–2.14) **	1.58 (1.23–2.04) ***
**Living situation**				
with other(s) at home	1.00	1.00	1.00	1.00
living alone	0.53 (0.39–0.72) ***	0.85 (0.68–1.08)	0.90 (0.65–1.25)	1.12 (0.86–1.46)
**Self-assessed health**				
good to very good health	1.00	1.00	1.00	1.00
fair, bad to very bad	1.0 (0.75–1.37)	1.45 (1.13–1.85) **	1.39 (0.97–1.98)	1.16 (0.88–1.54)
**Longstanding illness or health problem**				
No	1.00	1.00	1.00	1.00
Yes	1.28 (0.96–1.71)	1.36 (1.08–1.72) **	1.86 (1.31–2.65) ***	1.68 (1.28–2.21) ***
**BMI**				
<18.5	2.15 (1.06–4.37) *	0.96 (0.49–1.86)	0.36 (0.17–0.79) **	0.38 (0.20–0.75) **
18.5–25	1.00	1.00	1.00	1.00
25–30	1.28 (0.95–1.72)	1.45 (1.15–1.84) **	1.04 (0.74–1.45)	1.15 (0.88–1.51)
30+	1.04 (0.66–1.73)	1.28 (0.92–1.79)	1.11 (0.68–1.81)	0.98 (0.67–1.42)
**Smoking status**				
never	1.00	1.00	1.00	1.00
former	1.20 (0.86–1.67)	0.90 (0.69–1.18)	1.33 (0.90–1.96)	1.12 (0.83–1.53)
occasionally/daily	1.43 (0.92–2.21)	0.76 (0.54–1.08)	0.98 (0.60–1.60)	0.86 (0.59–1.12)
**Physical activity**				
≥30 min/day	1.00	1.00	1.00	1.00
<30 min/day	1.22 (0.83–1.79)	1.06 (0.80–1.40)	0.63 (0.41–0.95) *	0.81 (0.59–1.12)
**Highest level of education within the household**				
No degree or primary education	0.78 (0.51–1.19)	0.98 (0.70–1.37)	0.43 (0.26–0.71) ***	0.47 (0.32–0.70) ***
Lower secondary	0.91 (0.60–1.37)	1.03 (0.74–1.43)	0.60 (0.36–0.99) *	0.58 (0.39–0.87) **
Higher secondary	1.13 (0.77–1.66)	0.97 (0.71–1.33)	0.61 (0.37–1.01)	0.55 (0.38–0.81) **
Higher education	1.00	1.00	1.00	1.00
**Equivalent household income (€)**				
<750	3.03 (0.87–10.57)	1.18 (0.61–2.28)	1.08 (0.43–2.70)	1.27 (0.59–2.73)
750–1,000	3.40 (1.00–11.58) *	1.02 (0.54–1.92)	1.32 (0.53–3.25)	1.41 (0.67–2.96)
1,000–1,500	3.27 (0.98–10.88)	1.54 (0.83–2.83)	1.45 (0.60–3.46)	1.27 (0.62–2.60)
1,500–2,500	3.94 (1.16–13.33) *	1.21 (0.64–2.29)	1.22 (0.49–3.04)	1.23 (0.58–2.61)
>2,500	1.00	1.00	1.00	1.00

Notes: Complete case analyses, after adjustment for age, sex, region, living situation, risk factors and health status; Odds ratios (ORs); 95% confidence intervals (CIs); *p* values (Wald Chi-square test); * *p* ≤ 0.05; ** *p* ≤ 0.01; *** *p* ≤ 0.001.

The uptake of immunization among Belgian elderly respondents increases with age and female respondents are less likely to have had pneumococcus immunization and blood cholesterol measurement than male respondents. Belgian elderly respondents living alone are significantly less likely to report pneumococcus immunization, however, the use of other preventive services is not significantly linked with living situation. Belgian elderly with overweight are more likely to report an influenza immunization, and elderly with moderate physical activity per day (<30 min) are less likely to report a cholesterol measurement. We found no significant association between smoking status and preventive care use. Belgian elderly respondents who assess their health as moderate, bad to very bad are more likely to have had an influenza immunization. Elderly with longstanding illness or health problems are significantly more likely to have had blood cholesterol and blood sugar measurement, compared to those who without longstanding illnesses or health problems. Elderly respondents who assess their health as moderate, bad to very bad and elderly with longstanding illness or health problems are also significantly more likely to have had an influenza immunization.

After adjustment for socio-demographic factors, health status and risk factors, lower educated elderly are significantly less likely to have had a blood cholesterol and blood sugar measurement. Pneumococcus immunization was not significantly related to educational level, but elderly with an equivalent household income of €1500**–**2500 were more likely to have had a pneumococcus immunization compared with >€2500. Influenza immunization was not significantly related to SES. 

Our study indicates that there is no SE gradient in influenza immunization (before and after adjustment for needs). Still, a SE gradient in cholesterol and blood sugar measurement exists among the Belgian elderly population. Lower levels of education were significantly associated with lower preventive care use (except for influenza immunization). 

## 4. Discussion

An affordable healthcare system has always been an important element in Belgian health policy. The Belgian health system is based on the principle of social insurance characterized by vertical (proportional social security contributions related to income) and horizontal solidarity (contributions independent of risk) [43,44]. To protect the weakest SE groups, two important socially inspired measures to decrease financial barriers were developed and introduced by the Federal Governement: a “preferential rate” with reduced co-payments for specified vulnerable social categories including low-income pensioners and disability benefit recipients (in 2007 the OMNIO scheme extended the this increased reimbursement system to all low-income families) and a “maximum billing” (MAB) system which puts an upper limit (dependent on the net taxable household income) to the total amount of yearly co-payments for healthcare [43,44]. While many aspects of Belgian health policy are covered by federal authorities, some domains belong to the responsibilities of the regional governments. Preventive care is a regional responsibility, but the federal public health insurance provides reimbursements for immunizations and blood measurements. This situation potentially leads to differences in healthcare utilization by region. Healthcare utilization is also associated with environmental determinants such as the availability and accessibility of services, which can vary geographically. In Belgium, the density of practicing GPs and specialists varies between the regions [45]. This study, however, did not allow to explore this aspect further. Nevertheless, ‘region’ is included as an environmental factor, and adjustment is made for demographic and SE differences between the regions. Still, there are some significant regional differences in the uptake of preventive care among the Belgian elderly population. 

Almost the whole Belgian population (99%) is covered for a very broad benefits package [46]. However, compared with other European countries, ‘out of pocket’ payments are relatively high in Belgium [47,48]. Influenza and pneumococcus immunization often require three contacts with the healthcare system in Belgium: one with a GP to receive a prescription, one to buy the vaccine in a pharmacy and one to get the immunization. As in many other European countries, influenza and pneumococcus immunizations are still not fully covered by health insurance. How healthcare is financed may affect prevention uptake [13,14,49]. Carrieri [14] for instance, indicated that inequalities in blood tests are higher in countries with a high share of “out of pocket” payments. Our results confirm the study by Carrieri and Wuebker [14] who found for Belgium that—in comparison with many other European countries—after controlling for needs there is no pro-rich inequality in cholesterol and blood sugar measurement. However, lower education is significantly associated with lower preventive care use. There is no evidence that lower SE groups require less preventive care. On the contrary, lower SE groups have a greater risk for hypertension, diabetes, heart disease and need more preventive care [48].

Although lower SE groups in the Belgian elderly population use primary care more frequently than higher educated groups [2,50], and GP’s can offer lower SE groups to measure blood cholesterol and blood sugar (and pneumococcal immunization) more often, they still seem less likely to use such preventive care. Further research is needed to evaluate if the SE gradient in preventive care use increases after adjustment for GP contacts. 

Our results reveal a different impact of household income and educational level on preventive care use. Better educated individuals are assumed to choose healthcare inputs more efficiently, may have more access to regular preventive care, resources to overcome barriers, and may have more confidence to ask the GP directly about immunization or general health checks, because of their greater awareness of health risks and better understanding of health promotion messages [16]. Less educated people might have more problems in understanding the benefits of prevention than higher educated persons and consider treatments as unnecessary in the absence of symptoms and only seek treatment when health problems display symptoms [15]. Many people are not aware that influenza or pneumococcus immunization are recommended for them or even do not know that these interventions exist [21].

The GP is the most important source of encouragement for people to use appropriate preventive care interventions such as influenza and pneumococcus immunization or cholesterol measurement [14]. GPs can improve immunization uptake, but can also cause under-utilisation if they themselves are reluctant and unconvinced about the need for influenza and pneumococcus immunization [51]. Furthermore, lower SE groups in general receive less (cancer) screening recommendations by their GP [52]. Such inequality is unfair, as the opportunity of receiving the appropriate information is then dependent on the SE background of the patient. More often the GP initiates preventive care use, and not the patient. 

Higher educated groups might have a greater awareness of the specific preventive services they have had. This might be an explanation for the lack of SE gradient in influenza vaccination. Influenza immunization seems to be more known in the general Belgian population, compared with pneumococcus immunization. And, a GP might be less likely to inform lower educated patients about the different blood measurements that were conducted. 

An important strength of this study is the use of the Belgian HIS which is a large national population based sample that contains detailed data on health, SES and preventive care use. The HIS has a multistage sample design with stratification and systematic sampling to make the sample nationally representative, and has a relatively high participation rate. The use of multivariate logistic regression analyses leads to a meaningful estimate of the effect of SES, before and after adjustment for needs and risk factors. Some preventive services are recommended for specific groups, thus it makes little sense to measure SE inequalities in preventive care use without taking into consideration the distribution of needs and risk factors. 

Our study has some potential limitations. Firstly, self-reported data for measuring preventive care use can lead to under- or over-reporting of the use of preventive services. However, self-reported data on influenza immunization has been found to be highly sensitive [24]. Secondly, self-reported data may be subject to recall bias [26], especially when recall bias differs by SE groups [11]. This could be especially the case for pneumococcus immunization and blood cholesterol measurement where the time span comprised 5 years, and blood sugar measurement where the time span comprised 3 years. Furthermore, non-random misclassification could play a role. Higher educated are assumed to be more likely to report correct information regarding preventive care use. If information is more correct in higher SE groups compared with lower SE groups, the association may be over- or underestimated. Thirdly, preventive care use should target all individuals, regardless their health status, but some health conditions might require carrying out more prevention or are part of treatment. For cholesterol and blood sugar measurement no distinction can be made between screening for preventive purposes or for monitoring known health problems. However, we have excluded the majority of patients that get blood sugar measurement for treatment purposes by excluding glycaemia patients from our analyses. CVD are more prevalent among lower SE groups [53], thus it is logical that measurements for risk factors are more prevalent among these groups. But if this is the case, the results in this study may have concealed inequalities in the reverse direction in measurement undertaken for preventive purposes [29]. Finally, the cross sectional nature of the HIS prevents to draw conclusions about causal relationships. To explore causal relationships between SES and preventive care use, longitudinal data are preferable. 

## 5. Conclusions

There is no pro-rich inequality in influenza immunization and cholesterol and blood sugar measurement among the Belgian elderly population, after controlling for needs. Only elderly respondents with an equivalent household income of €1500**–**€2500 are significantly more likely to report pneumococcus immunization. However, lower educated groups are significantly less likely to report cholesterol and blood sugar measurement. 

Adjustment for socio-demographic factors, health status and risk factors does not influence the impact of SES significantly. However, the initially observed impact of educational level on pneumococcus immunization disappears after adjustment for all the determinants. The initial educational gradient can be explained by differences in health status or risk factors of the respondents. 

Our results highlight the need to promote cholesterol and blood sugar measurement for lower educated groups, and pneumococcus immunization for the entire elderly population. Influenza immunization seems to be equally spread among different SE groups. The results of our study may contribute to optimize preventive care use. It should be a goal of Belgian health policy to reduce health inequalities and to build conditions allowing equitable chances to everyone to benefit from preventive interventions such as pneumococcus immunization and cholesterol and blood sugar measurement. 

Belgian health policy seems rather effective as there is no significant lower use of preventive services among the lower income groups. However, lower levels of education were significantly associated with lower preventive care use (except influenza immunization).

## References

[1] Mackenbach J.P., Meerding W.J., Kunst A.E. Economic Implications of Socioeconomic Inequalities in Health in the European Union. http://ec.europa.eu/health/ph_determinants/socio_economics/documents/socioeco_inequalities_en.pdf.

[2] Hoeck S., François G., van der Heyden J., Geerts J., van Hal G. (2011). Healthcare utilisation among the Belgian elderly in relation to their socio-economic status. Health Policy.

[3] Hoeck S., François G., Geerts J., van der Heyden J., Vandewoude M., van Hal G. (2012). Health-care and home-care utilization among frail elderly persons in Belgium. Eur. J. Public Health.

[4] Lorant V., Boland B., Humblet P., Deliege D. (2002). Equity in prevention and health care. J. Epidemiol. Community Health.

[5] Cutler D.M., Lleras-Muney A. (2009). Understanding differences in health behaviors by education. J. Health Econ..

[6] Hunter D., Killoran A. (2004). Tackling Health Inequalities: Turning Policy into Practice?.

[7] Culica D., Rohrer J., Ward M., Hilsenrath P., Pomrehn P. (2002). Medical checkups: Who does not get them?. Amer. J. Public Health.

[8] Oxley H. (2009). Policies for Healthy Ageing: An Overview.

[9] Cohen J.T., Neumann P.J., Weinstein M.C. (2008). Does preventive care save money? Health economics and the presidential candidates. N. Engl. J. Med..

[10] Fineberg H.V. (2013). The paradox of disease prevention. Celebrated in principle, resisted in practice. JAMA.

[11] Patel R., Lawlor D.A., Ebrahim S. (2007). Socio-economic position and the use of preventive health care in older British women: A cross sectional study using data from the British women’s heart and health study cohort. Fam. Pract..

[12] Stirbu I., Kunst A.E., Mielck A., Mackenbach J.P. (2007). Educational Inequalities in Preventives Services Among Elderly in Europe. Tackling Health Inequalities in Europe: An Integrated Approach.

[13] Jusot F., Or Z., Sirven N. (2012). Variations in preventive care utilization in Europe. Eur. J. Ageing.

[14] Carrieri V., Wuebker A. (2013). Assessing inequalities in preventive care use in Europe. Health Policy.

[15] Schmitz H., Wübker A. (2011). What determines influenza vaccination take-up of elderly Europeans?. Health Economics.

[16] Carrieri V., Bilger M. (2013). Preventive care: Underused even when free. Is there something else at work?. Appl. Econ..

[17] Dalton A.R.H., Bottle R.A., Okoro C., Majeed F.A., Millett C. (2011). Uptake of the NHS health checks programme in a deprived, culturally diverse setting: Cross sectional study. J. Epidemiol. Community Health.

[18] Dryden R., Williams B., McCowan C., Themessl-Huber M. (2012). What do we know about who does and does not attend general health checks? Findings from a narrative scoping review. BMC Public Health.

[19] Jefferson T., Rudin M., di Pietrantonj C., Rivetti D., Rivetti A., Demicheli V. (2005). Efficacy and effectiveness of influenza vaccines in elderly people: A systematic review. Lancet.

[20] Christenson B., Hedlund J., Lundbergh P., Ortqvist A. (2004). Additive preventive effect of influenza and pneumococcal vaccines in elderly persons. Eur. Resp. J..

[21] Kohlhammer Y., Schnoor M., Schwartz M., Raspe H.T. (2007). Determinants of influenza and pneumococcal vaccination in elderly people: A systematic review. Public Health.

[22] Ward L., Draper J. (2007). A review of the factors involved in older people’s decision making with regard to influenza vaccination: A literature review. J. Clin. Nurs..

[23] Zimmerman R., Nowalk M.P., Tabbarah M., Hart J.A., Fox D.E., Raymund M. (2009). Understanding adult vaccination in urban, lower-socio-economic settings: Influence of physician and prevention systems. Ann. Fam. Med..

[24] Endrich M.M., Blank P.R., Szucs T.D. (2009). Influenza vaccination uptake and socioeconomic determinantsin 11 European countries. Vaccine.

[25] Chiatti C., di Rosa M., Barbadoro P., Lamura G., di Stanislao F., Prospero E. (2010). Letter to the editor: Socioeconomic determinants of influenza vaccination among older adults in Italy. Prev. Med..

[26] Steinberg D., Gotto A.M. (1999). Preventing coronary artery disease by lowering cholesterol levels: Fifty years from bench to bedside. J. Am. Med. Assn..

[27] Damiani G., Federico B., Bianchi C., Ronconi A., Basso D., Fiorenza S., Sassi F. (2011). Socio-economic status and prevention of cardiovascular disease in Italy: Evidence from a national health survey. Eur. J. Public Health.

[28] Ricci-Cabello I., Ruiz-Perez I., de Labry-Lima A.O., Marquez-Calderon S. (2010). Do social inequalities exist in terms of the prevention, diagnosis, treatment, control and monitoring of diabetes? A systematic review. Health Soc. Care Community.

[29] Rodin D., Stirbu I., Ekholm O. (2012). Educational inequalities in blood pressure and cholesterol screening in nine European countries. J. Epidemiol. Community Health.

[30] Bowden R.G. (2001). Comparisons of cholesterol screening participants and non-participants in a university setting. Int. Electron. J. Health Educ..

[31] Lairson D.R., Chan W., Newmark G.R. (2005). Determinants of the demand for breast cancer screening among women veterans in the United States. Soc. Sci. Med..

[32] Böhmer M.M., Walter D., Falkenhorst G., Müters S., Krause G., Wichmann O. (2012). Barriers to pandemic influenza vaccination and uptake of seasonal influenza vaccine in the post-pandemic season in Germany. BMC Public Health.

[33] Andrew M.K., McNeil S., Merry H., Rockwood K. (2004). Rates of influenza vaccination in older adults and factors associated with vaccine use: A secondary analysis of the Canadian study of health and aging. BMC Public Health.

[34] Shahrabani S., Benzion U. (2006). The effects of socioeconomic factors on the decision to be vaccinated: The case of flu shot vaccination. Isr. Med. Assoc. J..

[35] Lowensteyn I., Joseph L., Levinton C., Abrahamowicz M., Steinert Y., Grover S. (1998). Can computerized risk profiles help patients improve their coronary risk? The results of the coronary health assessment study (CHAS). Prev. Med..

[36] (2009). Vaccination Guide.

[37] Demarest S., van der Heyden J., Charafeddine R., Drieskens S., Tafforeau J. (2013). Methodological basics and evolution of the Belgian health interview survey 1997–2008. Arch. Public Health.

[38] Dalstra J.A., Kunst A.E., Mackenbach J.P. (2006). A comparative appraisal of the relationship of education, income and housing tenure with less than good health among the elderly in Europe. Soc. Sci. Med..

[39] Atkinson A.B., Rainwater C., Smeeding T.M. (1995). Income Distribution in OECD Countries: The Evidence from the Luxembourg Income Study (LIS). OECD Social Policy Study.

[40] Hagenaars A., de Vos K., Zaïdi M.A. (2001). Comparison Between Poverty Rates in Wave 1 ECHP for 1993.

[41] Braveman P.A., Cubbin C., Egerter S. (2005). Socioeconomic status in health research: One size does not fit all. JAMA.

[42] Oakes M.J., Rossi P.H. (2003). The measurement of SES in health research: current practice and steps toward a new approach. Soc. Sci. Med..

[43] Gerkens S., Farfan M.I., Desomer A., Stordeur S., de Waroux M., van de Voorde C., van de Sande S., Leonard C. (2010). The Belgian Health System in 2010.

[44] Corens D. (2007). Belgium: Health system review. Health Syst. Transit..

[45] Roberfroid D., Stordeur S., Camberlin C., van de Voorde C., Vrijens F., Léonard C. (2008). Physician Workforce Supply in Belgium: Current Situation and Challenges.

[46] (2009). Statistics and Indicators for 30 Countries.

[47] Or Z., Jusot F., Yilmaz E. Impact of Health Care System on Socioeconomic Inequalities in Doctor Use. http://www.irdes.fr/EspaceAnglais/Publications/WorkingPapers/DT17ImpactHealthCareSystSocioeconomicInequalities.pdf.

[48] Adler N.E., Newman K. (2002). Socioeconomic disparities in health: Pathways and policies. Health Affair..

[49] Or Z., Jusot F., Yilmaz E. (2009). The European Union Working Group on Socioeconomic Inequalities in Health. Inégalite’s Sociales de Recours aux Soins en Europe: Quel Rôle Pour le Système de Soins?. Revue. Econ..

[50] Hoeck S., van der Heyden J., Geerts J., van Hal G. (2013). Equity in GP and specialist contacts by older persons in Belgium. Int. J. Public Health.

[51] Nichol K.L., Zimmerman R. (2001). Generalist and subspecialist physicians’ knowledge, attitudes, and practices regarding influenza and pneumococcal vaccinations for elderly and other high-risk patients: A nationwide survey. Arch. Intern. Med..

[52] O’Malley M., Earp J.A., Hawley S.T., Schell M.J., Mathews H.F., Mitchell J. (2001). The association of race/ethnicity, socioeconomic status, and physician recommendation for mammography: Who gets the message about breast cancer screening?. Amer. J. Public Health.

[53] Dalstra J.A., Kunst A.E., Borrell C. (2005). Socioeconomic differences in the prevalence of common chronic diseases: An overview of eight European countries. Int. J. Epidemiol..

